# What are the learning objectives in surgical training – a systematic literature review of the surgical competence framework

**DOI:** 10.1186/s12909-024-05068-z

**Published:** 2024-02-06

**Authors:** Niklas Pakkasjärvi, Henrika Anttila, Kirsi Pyhältö

**Affiliations:** 1 Department of Pediatric Surgery, New Children’s Hospital, Helsinki University Hospital, Helsinki, Finland; 2 Department of Pediatric Surgery, Section of Urology, University Children’s Hospital, Uppsala, Sweden; 3 Faculty of Educational Sciences, University of Helsinki, Helsinki, Finland; 4 Centre for Higher and Adult Education, Faculty of Education, Stellenbosch University, Stellenbosch, South Africa

**Keywords:** Surgical competence, Surgical education, Systematic literature review

## Abstract

**Objective:**

To map the landscape of contemporary surgical education through a competence framework by conducting a systematic literature review on learning outcomes of surgical education and the instructional methods applied to attain the outcomes.

**Background:**

Surgical education has seen a paradigm shift towards competence-based training. However, a gap remains in the literature regarding the specific components of competency taught and the instructional methods employed to achieve these outcomes. This paper aims to bridge this gap by conducting a systematic review on the learning outcomes of surgical education within a competence framework and the instructional methods applied. The primary outcome measure was to elucidate the components of competency emphasized by modern surgical curricula. The secondary outcome measure was to discern the instructional methods proven effective in achieving these competencies.

**Methods:**

A search was conducted across PubMed, Medline, ProQuest Eric, and Cochrane databases, adhering to PRISMA guidelines, limited to 2017–2021. Keywords included terms related to surgical education and training. Inclusion criteria mandated original empirical studies that described learning outcomes and methods, and targeted both medical students and surgical residents.

**Results:**

Out of 42 studies involving 2097 participants, most concentrated on technical skills within competency-based training, with a lesser emphasis on non-technical competencies. The effect on clinical outcomes was infrequently explored.

**Conclusion:**

The shift towards competency in surgical training is evident. However, further studies on its ramifications on clinical outcomes are needed. The transition from technical to clinical competence and the creation of validated assessments are crucial for establishing a foundation for lifelong surgical learning.

## Introduction

 Surgery requires a highly specialized set of surgical knowledge, skills, and attitudes that will allow a surgeon to perform the requisite procedures in collaboration with the patient and the multi-professional team. These competencies are fundamental to a surgeon’s ability to function effectively, necessitating flexibility, adaptability, and continuous professional development. In the field of learning sciences, the term competence is used to refer to the combination of knowledge, skills, and attitudes that allows an individual to solve the job-related task or a problem at hand and act professionally [1–4]. Accordingly, it can be claimed that cultivating a set of surgical competencies organically integrating knowledge, skills, and attitudes needed in surgeons’ work is imperative for high-quality surgical education. This calls for the understanding of both the range of competencies acquired in surgery training and the kinds of instructional methods that are effective in adopting them. Interestingly, many studies in surgical education, including systematic literature reviews, appear to often focus on a single learning outcome. This typically involves exploring either a specific technical skill or content knowledge in a surgical area, along with assessing the effectiveness of a particular instructional method [5–9].

The traditional Halstedian methods, with their focus on incremental responsibility and volume-based exposure, have been foundational in surgical training. Over the past few decades, the approach has been complemented with more tailored instructional methods [10, 11]. For example, technical skills are often contemplated with models and simulators [12, 13], thus increasing patient safety during surgery, and allowing the training surgeon to focus on the operation without feeling pressured to execute technical tasks [11]. Simulation training has demonstrated positive effects, especially in technical skills [14–16], but also in the longitudinal transfer of skills [17]. Much of the research on simulation has focused on training assessment with validated programs becoming more widely available [18–22]. Procedure-specific assessment has become common in evaluating surgical learning outcomes and has resulted in a set of validated task-specific assessment tools, such as OSATS (Objective Structured Assessment of Technical Skills) [23]. However, reducing surgery to separated technical tasks infers risks related to developing surgical competence, mainly a lack of integration in the learning of surgical skills, knowledge, and attitudes, further compromising continuous professional development, and thus potentially occupational wellbeing. There is also contradictory evidence on the effectiveness of the surgical training method in achieving the desired learning outcomes, but this may be more related to the unrealized potential of evidence-based training methods [24]. Further, the implementation of modern surgical training is lagging [25]. To sum up, while research on surgical education has significantly advanced our understanding of more tailored methods for cultivating surgical learning, it has also typically adapted a single ingredient approach [10, 11]. A problem with this approach is that it neglects the complexity of surgical competence development and, without coherence building, bears the inherent risk of reducing surgery into mastering a series of technical tasks rather than providing tools for cultivating surgical competencies. Moreover, only a few prior systematic reviews on surgical education have studied surgical learning across the fields of surgery or among both medical students and surgical residents. Our study aims to comprehensively analyze the competencies targeted in contemporary surgical education, as revealed through a systematic literature review. We seek to elucidate the nature of these competencies—including skills, knowledge, and attitudes—and the instructional methods employed to develop them in medical students and surgical residents. This approach will highlight how competencies are defined, integrated, and cultivated in surgical education according to existing literature. Specifically, our primary outcome is to identify and detail the competencies (skills, knowledge, and attitudes) emphasized in the existing research on surgical education. We aim to understand how these competencies are conceptualized, taught, and developed, providing insights into the current focus of surgical training programs. As a secondary objective, we will examine the instructional methods discussed in the literature for teaching these competencies. This involves analyzing the effectiveness and application of different teaching strategies in nurturing a comprehensive set of surgical competencies, focusing on integrating technical and non-technical skills. To our knowledge, this is the first published effort within surgery to review the literature comprehensively on surgical competencies development and instructional methods across the fields of surgery, with studies conducted with both medical students and surgical residents.

## Methods

We conducted a systematic literature review by using the guidelines of the Preferred Reporting Item for Systematic Reviews and Meta-analysis statement (PRISMA) [26].

### Research strategy and data sources

 We searched four electronic databases: PubMed, Medline, ProQuest Eric, and Cochrane databases on 18 February 2021. Only articles in English were considered, and the search was limited to years 2017–2021. This restriction was based on a pilot search, which identified a high volume of review articles before 2017 and a significant increase in the quantity and relevance of primary research studies on the surgical competence framework beginning in 2017. The search string consisted of the following keywords: “Surgical Education”, “Surgical Training”, “Surgical Intern*”, “surgical resident” OR “surgical apprentice” AND “learning”. The detailed syntax of the search was: (“surgical intern” AND learning) OR (“surgical training” AND learning) OR (“surgical intern*” AND learning) OR (“surgical resident” AND learning) OR (“surgical apprentice” AND learning). The database search resulted 1305 articles (1297 from PubMed/Medline, 6 from Cochrane databases, and 2 from ProQuest Eric).

### Inclusion criteria and study selection

We applied five inclusion criteria for the data. To be included in the review, the articles had to fulfil the following criteria:

- be original empirical studies.
- be published in a peer-reviewed journal between 2017 and 2021.
- be written in English, although the study could have been conducted in any country.
- include surgical residents and/or medical students as participants.
- include descriptions of learning outcomes and methods of learning in the results of the study.

**Fig. 1:**
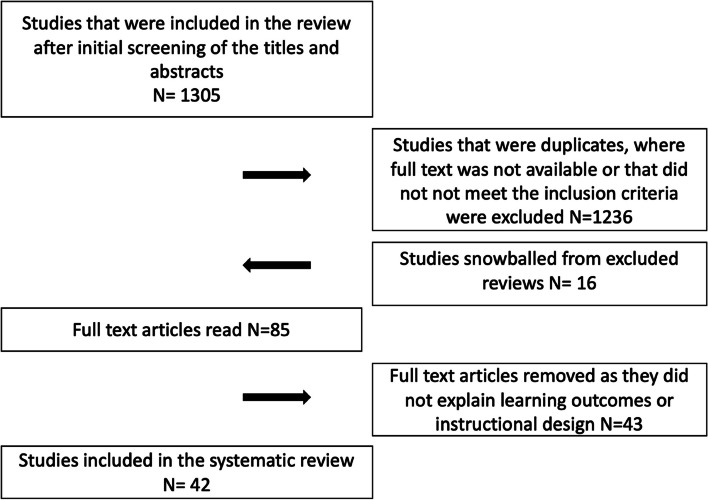
The PRISMA diagram depicts the flow of the systematic review, from the initial identification of 1305 database hits to the ultimate inclusion of 42 articles

Data were extracted manually in several increments. Two of the authors (NP) and (HA) independently reviewed the titles and abstracts of all articles identified by the search and marked potentially relevant articles for full-text retrieval (see Fig. 1 for the PRISMA diagram for the review flow). After reading the titles and abstracts, and removing the duplicates, 1236 articles were excluded as they did not meet the inclusion criteria. This also included 13 literature reviews that were excluded from the study as they were not empirical. However, the references of the reviews were reviewed by using a snowball method to detect additional references. This resulted in 16 studies being added to the full-text analysis. After this, the two authors independently examined the full texts of the remaining 85 articles with the inclusion criteria and selected the studies eligible for inclusion in the review. At this point, 43 articles were excluded as they did not explain learning outcomes or learning activities. Disagreements between the two authors were minimal and were resolved through a joint review of the full-text articles and discussion with the third co-author (KP). All articles that matched the inclusion criteria were included in the review, resulting in 42 articles being included in the review.

### Data extraction

Two of the authors (NP) and (HA) extracted and documented information about 11 factors of each study into the Excel file to create a data sheet for the analysis. The following characteristics of the studies were recorded: country, participants, field of surgery, study design, use of a control group, tool, outcome measure, core finding, results on surgical learning outcomes, instructional design applied and clinical setting. *Learning outcomes* were categorized according to the three components of surgical competence: (a) *knowledge*, (b) *skills* (including both technical and non-technical skills), and (c) *attitudes* [22]. *Surgical knowledge* included results concerning training surgeons’ theoretical and practical knowledge about surgery, procedure, or medicine in more general. *Surgical skills* entailed results on their technical and non-technical skills, strategies, reflection, and self-regulation. *Surgical attitudes* involved results on training surgeons about their attitudes to their work and views about themselves as surgeons. The instructional design reported in the studies was coded into seven categories according to the mode of instruction applied in the study for training surgeons: (a) *learning by doing*, including (b) *learning through reflection*, including instructions where the training surgeons reflected their own learning (c) *learning by modelling*, (d) *learning by direct instruction*, (e) *learning by self-directed study*, (f) *learning by mentoring or teaching*, and *(g) learning by gaming.*

The “*Learning by doing*” category included instructional situations in which medical students and surgeons learned while working as surgeons, for example, by completing surgical tasks and procedures. “*Learning through reflection*” included situations in which they learned by reflecting on their prior experiences, thoughts, own development, and performance in specific tasks.

In the “*Learning by modeling*” category, learning occurred by observing or copying the behaviors of their peers or more experienced surgeons. “*Learning by direct instruction*” included situations in which they learned while attending formal education, lectures, or seminars and by receiving tips or practical guidance from others.

The “*Learning by self-directed study*” category encompassed situations where training surgeons learned through self-directed study, such as reading, seeking information, and independently watching procedure videos, without any external intervention.

In the “*Learning by mentoring or teaching*” category, training surgeons learned while they taught or mentored their peers. “*Learning by gaming*” included situations where training surgeons played games to improve their competence.

Regarding categorization, each of the studies included in the review could belong to one or more of these categories. However, to be included in a category, the article needed to clearly explain that the instructional method in question was used in the study. For example, even though performing surgical procedures might also involve self-reflection, the article was categorized under “*learning by doing*” and not additionally under “*learning by self-reflection*” unless the reflection was explicitly mentioned in the article.

## Results

We included 42 empirical studies involving 2097 medical students and surgeons in training in this systematic review. The studies on surgical learning were geographically distributed across ten countries. Most of the studies were conducted in the USA (*n* = 22), and Canada (*n* = 12), however studies from the UK, the Netherlands, Austria, Chile, Germany, Finland, and Switzerland were also present. Surgical learning was typically explored with small-scale studies with a median of 28 participants, interquartile range 46 (see Table 1). Most of the studies focused on surgical residents’ learning (*n* = 29), whereas medical students’ surgical learning was explored in 11 studies. One study had both residents and medical students as participants. Twenty-seven studies investigated surgical learning in general surgery, with the remaining 16 in various other surgical specialties (including gynecology, cardiology, urology, pediatrics, neurosurgery, microsurgery, orthopedics, vascular surgery, gastro surgery and otolaryngology). The study design of the empirical studies varied from simulation (including bench models, animals, human cadavers, and virtual reality (VR)), operating room (OR) procedures, interviews, surveys, writing tasks, to knowledge tests and the resident report card. Most of the studies employed multimodal designs. Eighteen of the studies were controlled; 13 studies were randomized controlled trials (RCT), and five were controlled trials (CT). The core finding was discussed in all studies and where applicable, statistical tests were applied to highlight the significance. Almost half of the studies (*n* = 18) were conducted in clinical settings.

**Table 1:** Modes of surgical learning in the included studies

Author, year	Participants	Field of surgery	Study design	Control group	Tool	Outcome measure	Core finding	Learning outcomes	Instructional design	Clinical setting Yes/No
Babchencko et al. (2020) [27]	Residents, *N* = 134	General	Survey	n/a	Self-report, performance assessment	Questionnaire, Qualitative content analysis	Positive changes during residency included increased confidence (50%), improved technical skills (32%), and increased resilience (26%).	Technical skills, attitudes	Learning by doing	Yes
Geoffrion et al. (2019) [28]	Residents, *N* = 46	Gynecology	Knowledge test, Survey	RCT	Self-report, performance assessment	Knowledge test. Validated Self-Confidence Scale	Knowledge improved significantly in the intervention group and for each module tested. Self-confidence in the actual OR was only improved in the most novice trainees and for the most high-stake procedures	Attitudes, knowledge	Learning by doing, learning by self-directed study	Yes
Korte et al. (2020) [29]	Students and residents, *N* = 13	Cardiothoracic	Simulation (bench model and animal)	CT	Performance assessment (other report), self-report	Multidimensional skill matrix Scoring matrix based on OSATS	Deliberate practice improves performance	Technical skills	Learning by doing	No
Pandian et al. (2017) [30]	Residents, *N* = 26	General	Simulation (bench model)	CT	Performance assessment	Surgical Olympics objective assessment score	home-based curriculum before surgical residency, improved performance on early knowledge assessments. No difference in technical skills.	Technical skills, knowledge	Learning by modelling, learning by instruction, learning by doing	No
Charak et al. (2020) [31]	Student, *N* = 128	General	Knowledge test, Survey	CT	Self-report, Performance assessment	National exam	The students receiving the longitudinal curriculum outperformed the block students on the national shelf exam The longitudinal students were also more likely to learn directly from attending surgeons	Non-technical skills, knowledge	Learning by doing, learning by modelling	Yes
Harriman et al. (2019) [32]	Residents, *N* = 27	Urology	The resident report card	n/a	Performance assessment (other report), self-report	OSATS based objective competency assessment	Standardized assessment can monitor performance over time and is beneficial for learning.	Technical skills	Learning by reflection, learning by doing	Yes
Kumins et al. (2021) [33]	Student, *N* = 221	General	Knowledge test, survey,	n/a	Performance assessment, self-report,	MCQ knowledge test and objective skill exam (GRS)	Self-directed progressive video training is effective and improves technical skills and knowledge	Technical skills, knowledge	Learning by modelling, self-directed study	No
Peshkepija et al. (2017) [34]	Residents, *N* = 120	General	Writing task	n/a	Self-report	Qualitative content analysis, Summary statistics	Meaningful self-reflection was initially uncommon among all residents, but we were able to more than double this across the consortium by continuous process improvement.	Non-technical skills	Learning by reflection	Yes
Cadieux et al. (2021) [35]	Residents, *N* = 20	General	Interview	n/a	Self-report	Qualitative analysis	Residents used a range of preoperative preparatory strategies addressing four areas of focus: develop technical skills, improve procedural knowledge, enhance patient-specificity, and know surgical preferences	Technical skills, knowledge	Learning by self-directed study	Yes
Dressler et al. (2018) [36]	Student, *N* = 56	General	Writing task	n/a	Self-report	Qualitative analysis	Students could reflect on their experiences. Short format writing can be an effective format for reflection	Non-technical skills	Learning by reflection	Yes
Feins et al. (2017) [37]	Residents, *N* = 27	Cardiac	Simulation (animal, bench model)	n/a	Performance assessment (other report)	OSATS based assessment tool	Performance in component tasks and complete cardiac surgical procedures improved by simulation.	Technical skills	Learning by doing	No
Patel et al. (2018) [38]	Residents, *N* = 15	General	Inter-view	n/a	Self-report	Qualitative analysis	Impression management strategies were used to portray an image of competence, with the aim to improve access to teaching and evaluations. Unintended consequences of impression management on decision making, patient safety, and resident wellness were identified., patient safety and resident wellness.	Non-technical skills	Learning by direct instruction	Yes
Acosta et al. (2018) [39]	Residents, *N* = 20	General	Survey	n/a	Self-report	Survey of technical skills, urgent patient management, administrative tasks, and knowledge	Practical skills curriculum Significant differences were found over time in interns’ perceptions on their technical skills, patient management, administrative tasks, and knowledge	Technical skills, knowledge, attitudes,	Learning by doing	No
Chon et al. (2019) [40]	Student, *N* = 140	General	Knowledge Test	CT	Performance assessment	knowledge (measured with multiple choice questions and with written questions derived from an Objective Structured Clinical Examination station	A pre-test / post-test comparison yielded a significant increase in declarative knowledge.	Knowledge	Learning by gaming	No
Green et al. (2018) [41]	Residents, *N* = 32	General	Simulation (bench model)	n/a	Performance assessment (other report)	OSATS based assessment	preparatory courses improve performance and technical skills, and the skills are maintained upon matriculation into surgical residency.	Technical skills	Learning by doing	No
Hancock et al. (2021) [42]	Residents, *N* = 24	General	Knowledge test	n/a	Performance assessment	ABSITE scores	ABSITE scores improve by implementation of surgical jeopardy.	Knowledge	Learning by gaming	No
Jokinen et al. (2019) [43]	Residents, *N* = 20	Gynecology	Simulation (bench model)	RCT	Performance assessment (other report)	NRS, OSATS, OSA-LS	No differences in operative time, blood loss, or complications, nor in OSATS or NRS scores. In the intervention group, participants with the weakest performances in the simulator, seemed to benefit from the training program more than the participants with the best performances	Technical skills, knowledge	Learning by direct instruction, learning by doing	Yes
Zundel et al. (2017) [44]	Residents, *N* = 65	Pediatric	Survey	n/a	Self-report	Quantitative analysis	Residents learn from each other on wards and in the emergency department. They ranked fellow residents as first or second most important source of instruction.	Non-technical skills, knowledge	Learning by teaching or mentoring	Yes
Bohl et al. (2019) [45]	Residents, *N* = 12	Neurosurgery	simulation (bench model), survey	n/a	Self-report, performance assessment (other report)	Quantitative analysis, NASA TLX Scores	Resident and attending neurosurgeons subjectively believe that high-fidelity synthetic models were superior to cadavers as a surgical skill teaching platform.	Technical skills, knowledge	Learning by doing	No
Lees et al. (2019) [46]	Residents, *N* = 7	General	Interview	n/a	Self-report,	Qualitative analysis	Resident confidence was influenced by internal and external factors operating before, during, and after surgical task.	Non-technical skills, attitudes	Learning by doing, Learning by reflection	Yes
Harris et al. (2018) [47]	Students, *N* = 90	General	Simulation (robotic)	RCT	Performance assessment (other report)	Assessment; time, number of errors, rate of errors, smoothness	findings suggest that 3D observation provides no additional benefit to skill learning above 2D observation for early-stage robotic skills.	Technical skills	Learning by modelling	No
Gabrysz-Forget et al. (2020) [48]	Residents, *N* = 206	General	Survey	n/a	Self-report	Qualitative analysis and quantitative analysis.	Current teaching on error recovery perceived to be inadequate by learners. Trainees reported learning to recover from error through observation Only 37% felt they were adequately trained for error recovery.	Non-technical skills, attitudes	Learning by doing, learning by reflection	Yes
Klitsie et al. (2017) [49]	Student, *N* = 112	General	Knowledge test	RCT	Performance assessment	Pre and post Anatomy test of inguinal region in anatomical model	Dissection based training yielded superior scores on anatomy tests. Results were maintained medium-term.	Knowledge	Learning by doing	No
Siroen et al. (2017) [50]	Residents, *N* = 42	General	Simulation (robotic)	RCT	Performance assessment	(Robotic trainer w. corresponding tests)	Block training does not seem more effective than random training for robotic dry-lab skills	Technical skills	Learning by doing	No
Maertens et al. (2017) [51]	Residents, *N* = 32	General	OR procedures	RCT	Performance assessment (also other report)	MCQ cognitive endovascular skills, technical skill assessment (objective), objective evaluation of clinical procedure	Trainees who completed PROSPECT showed superior technical performance compared with trainees receiving e-learning alone or traditional education.	Technical skills	Learning by doing, learning by instruction, learning by self-directed study,	Yes
Raîche et al. (2019) [52]	Residents, *N* = 18	General	Interview	n/a	Self-report	Qualitative analysis	Surgical observation is perceived by residents as a learning activity with rich potential. Residents are challenged by focusing attention on most pertinent element and understanding what is occurring	Knowledge	Learning by modelling	Yes
LeCompte et al. (2019) [53]	Student, *N* = 16	General	Simulation	RCT	Performance assessment	OSATS based, physical measurements of anastomosis	Progressive autonomy in training yielded faster operative times, but no differences in final technical scores could be detected.	Technical skills	Learning by doing, learning by direct instruction	No
Kinio et al. (2019) [54]	Student, *N* = 13	Vascular Surgery	Simulation, Survey	n/a	Performance assessment, self-report	Quantitative analysis, completion of task	83% were motivated to prepare beforehand. 77% enjoyed the practical experiences and 54% preferred the inclusion of Escape rooms into medical curriculum.	Technical skills, knowledge, attitudes	Learning by gaming	No
Soucisse et al. (2017) [55]	Residents, *N* = 28	General	Simulation (animal)	RCT	Performance assessment	OSATS	Surgical video coaching led to increased technical scores. Video coaching is effective in this setting.	Technical skills	Learning by doing, learning by reflection	No
Ranney et al. (2021) [56]	Residents, *N* = 14	General	Interview	n/a	Self-report	qualitative analysis	Intra and interoperative phases of learning were identified. Self-reflection and awareness ability improves OR learning.	Knowledge, non-technical skills	Learning by reflection, learning by self-directed study	Yes
Naik et al. (2018) [57]	Students, *N *= 56	General	Simulation (bench model)	CT	Performance assessment (other report)	completion(yes/no), time to completion, OSATS	Video-feedback during technical skill learning improves performance in objective testing	Technical skills	Learning by doing, learning by direct instruction, learning by reflection	No
Lesch et al. (2020) [58]	Students, *N* = 37	General	Knowledge tests, survey	RCT	Performance assessment, self-report	Knowledge tests, quantitative analysis	Video and simulator enhanced various aspects of learning. Participants felt more confident in their ability to verbally reproduce procedural steps after using the simulation, but this did not translate directly into improved post quiz scores.	Technical skills, knowledge, attitude	Learning by doing, learning by reflection	No
Fletcher et al. (2020) [59]	Residents, *N* = 17	Vascular surgery	Simulation (bench model), knowledge tests, survey	n/a	Performance assessment, self-report	pre- and post-test, quantitative analysis	The use of a low-cost simulator increased procedure-specific knowledge, comfort, and confidence.	Knowledge, attitudes	Learning by doing,	No
Lee-Riddle et al. (2020) [60]	Residents, *N* = 31	General	Simulation (bench model) Survey	n/a	Self-report	Quantitative analysis	Boot camp increased confidence, follow-up demonstrated no difference in confidence compared to post boot camp results.	Attitudes	Learning by doing	No
Amer et al. (2017) [61]	Student, *N* = 100	General	Knowledge test, Simulation (VR), survey	RCT	Performance assessment, self-report	standardized test, Qualitative analysis	Students utilizing the app outperformed the control group on a standardized test.	Knowledge	Learning by gaming	No
Bhattacharyya et al. (2017) [62]	Residents, *N* = 16	Orthopedic	Simulation (bench model)	RCT	Performance assessment (other report)	objective assessment scores + subjective Likert scale	CTA utilization improved objective test scores significantly.	Knowledge, technical skills	Learning by direct instruction,	No
Levin et al. (2018) [63]	Residents, *N* = 19	Orthopedic	Simulation (bench model) knowledge test, survey	n/a	Performance assessment, Self-report	Quantitative analysis	App was believed by subjects to improve understanding and accelerate learning. CTA based app	Knowledge	learning by doing	No
Logishetty et al. (2020) [64]	Residents, *N* = 36	Orthopedic	Simulation (VR) knowledge test	RCT	Performance assessment	The number of times an incorrect next step was attempted, or an incorrect instrument was selected, the number of times assistance was required from the investigator, the time (minutes) taken to perform the THA, acetabular orientation error (°), and the knowledge test score (out of 10).	Preparing for arthroplasty by CTA-tool improved operative times and reduced mistakes.	Knowledge, technical skills	Learning by self-directed study, learning by doing	No
Trickey et al. (2017) [65]	Residents, *N* = 25	General	Survey, Simulation	n/a	Performance assessment (other report), self-report	SP-CAT	Residents’ confidence increased and their communication skills improved	Non-technical skills, attitudes,	Learning by reflection	Yes
Grant et al. (2017) [66]	Residents, *N* = 8	Microsurgery	Simulation (bench model) Survey	n/a	Performance assessment (other report), self-report	UWOMSA	Self-assessment scores correlate with preceptor scores. Learners with greater experience (higher postgraduate year level) tended to have higher self as well as preceptor ratings	Technical skills, non-technical skills	Learning by reflection	Yes
Quick et al. (2017) [67]	Residents, *N* = 14	Gastro surgery	OR procedure	n/a	Performance assessment (other report), self-report	OSATS	Junior residents believe they are more skilled technically; however, they maintain a humble attitude toward less technical aspects, such as operative flow, knowledge, and overall comfort.	Technical skills, non-technical skills, knowledge	Learning by doing, learning by reflection	Yes
Jethwa et al. (2018) [68]	Residents, *N* = 16	Otolaryngology	Simulation (human cadaveric)	RCT	Performance assessment (other report), self-report	Task-based checklist and global rating scale	One session of video self-review did not improve competence over standard practice.	Technical skills	Learning by doing, learning by reflection	No

### Primary outcome measures: learning objectives of surgeons in training and competency components

Most of the included studies on surgical learning focused on surgical skills and their attainment (*n* = 36) (See Table 1). Training surgeons commonly learned technical skills such as knot tying, distinct surgical procedures, and robotic skills (*n* = 25). In contrast, learning of non-technical skills (*n* = 11), such as communication, patient management, reflection, self-regulation, and decision-making skills, were less often reported. Twenty-two studies focused on the acquisition of surgical knowledge, such as general medical or surgical knowledge or more specific knowledge of certain procedures. Some of the studies (*n* = 10) reported attitudinal learning outcomes including confidence, resilience, and self-efficacy. Most of the studies (*n* = 26) had a single focus on surgical competence, i.e., they focused on learning of skills, knowledge, or attitudes. However, in 19 studies, the training surgeons’ learning was a combination of several skills, knowledge, and attitudes, most typically technical skills, and surgical knowledge. Empirical studies relied on performance assessment (*n* = 15), including studies in which the performance assessment was utilized by other reports, such as senior surgeons assessing the performance of the training surgeons, and self-reporting of the learning outcomes (*n* = 11). Sixteen studies combined both performance assessment and self-report of learning.

Learning was measured with validated objective tools in half of the studies. Most studies utilized either the OSATS global evaluation tool or a derivative optimized for the given conditions. These derivatives included ABSITE (The American Board of Surgery In-Service Training Exam) [69]; OSA-LS (OSATS salpingectomy-specific form) [70]; ASSET (Arthroscopic Surgical Skill Evaluation Tool) [71]; SP-CAT (Simulation Participants-Communication Assessment Tool) [72]; UWOMSA (University of Western Ontario Microsurgical Acquisition/assessment instrument) [73], and NRS (Numeric Rating Scale). Cognitive task analysis (CTA) was utilized in only two studies. In both studies, CTA improved scores in outcome testing [62, 64]. CTA-based training was considered suitable for expediting learning but based on our study cohort, it is scarcely applied.

### Secondary outcome measures: what kind of instructional designs do surgeons in training learn through?

The included studies in the present review employed various instructional methods ranging from learning by doing to mentoring and teaching fellow residents. *Learning by doing*, including technical training (of specific procedures, knot tying, etc.) both in OR settings and in simulation (e.g., VR, robotic, bench model, human cadaver, and animal), was most typically applied as the primary instructional method (*n* = 26), especially in teaching technical skills and non-technical surgical skills both for surgical residents and medical students. Partly mixed resulted in terms of the effectiveness of the method for novice and more advanced surgical students. For example, while Feins et al. showed that residents’ performance in component tasks and complete cardiac surgical procedures improved by simulation, Korte et al. reported, that especially more novice surgeons benefitted from simulations more than those who had more experience [29, 37]. Most skill curricula improved assessment scores, but surgical outcomes may remain unaffected by similar interventions as shown by Jokinen et al. [43]. Also, *learning through reflection*, through which training surgeons reflecting on their own learning experiences and development, such as by participating in debriefing after operations or via video-based guided reflection (*n* = 13) was a commonly emphasized instructional method. Engaging in reflection was shown to be effective in promoting the learning of non-technical skills and attitudes. Trickey et al. showed that reflecting on positive learning experiences increased residents’ confidence and improved their communication skills, while Soucisse et al. and Naik et al. reported that self-reflecting on surgical tasks performed improved technical skills as well [55, 57, 65]. Ranney et al. furthermore showed that residents, who can reflect on their learning and thought processes are more in control and proceed to autonomy more quickly [56].

Commonly used instructional methods for enhancing surgical learning include *modeling* (*n* = 5), particularly observing more experienced surgeons performing surgical procedures, s*elf-directed study* (*n* = 6), such as preparing for surgery, reading, and self-studying and *direct instruction* (*n* = 7). The latter included participating in contact teaching and lectures, watching videos, and getting practical advice from senior surgeons, and these were frequently used in teaching future surgeons. Raiche et al. showed that observing and modelling, have their limitations, as residents have challenges in identifying where to focus their attention and in understanding what it is teaching them [52]. To be effective, such a form of instruction seems to call for explanation and support from senior surgeons. Naik et al. showed that receiving feedback during technical skill learning had a significant impact on residents’ performance in technical skills [57]. The results also emphasized the importance of pre-preparation for the OR for learning gains. For example, Logishetty et al. showed that residents preparing for arthroplasty with a CTA tool improved operative times and reduced mistakes and were taught both decision-making skills as well as technical skills [64].

On the other hand, *learning through gaming* (including playing escape rooms, jeopardy, and other quiz games) (*n* = 4) and *mentoring or teaching fellow training surgeons* (*N* = 1) were seldomly applied in the teaching of future surgeons. The empirical evidence still implies that such instructional methods can enhance surgical learning. Hancock et al., Chon et al., Kinio et al. and Amer et al., all showed that gaming improved surgical knowledge [40, 42, 54, 61]. Zundel et al. found that peers are an extremely important source of instruction for training surgeons and that they both acquire knowledge and learn technical skills every day from each other [44]. Unfortunately, they receive little educational training in peer mentoring and thus the resource of peers as learning support is not exploited to its full potential [44].

To sum up, the results indicate that multimodal instructional designs are more commonly applied in studies exploring surgical learning and means to enhance it. In just over half of the studies (*n* = 23) participants were engaged in a combination of two to three different instructional activities.

## Discussion

Our results show that studies on surgical residents and medical students’ surgical learning focus heavily on learning surgical skills, particularly technical skills, and acquiring knowledge on how to perform specific procedures or surgical tasks. This indicates that, at least implicitly, quite a few studies on surgical learning are drawing on a competence framework by combining the learning of surgical skills and knowledge acquisition. However, the scope of such studies typically remains very specific.

Learning surgical soft skills such as communication and teamwork, learning skills, and adaptability were rarely investigated. Interestingly, none of the studies address learning skills such as self- or co-regulated learning as part of surgical learning. However, they are fundamental for flexible and adaptive professional behaviors and engagement in continuous professional development [74, 75]. In addition, the studies included in the review rarely addressed learning of attitudes such as self- or co-efficacy or resilience as part of surgical learning, though self-efficacy has shown to be one of the main predictors of learning outcomes and good performance [76, 77]. This may imply that such skills and attitudes are not considered to be at the core of surgical learning or that they are expected to result as by-product of other surgical learning activities. This can be considered to be a gap in the literature on surgical learning. The lack of knowledge on developing soft skills and attitudes among future surgeons also has practical implications since they play a central role in patient safety and a surgeon’s recovery from adverse events [78, 79]. The importance of these non-technical skills is further supported by research from Galayia et al. and Gleason et al. [80, 81]. Their studies highlight how factors like workload, emotional intelligence, and resilience are crucial in managing burnout, with a clear correlation shown between these skills, job resources, and burnout rates among surgical trainees.

Surgeons’ lack of familiarity with non-technical skills and insufficient training for handling adverse events [82, 83] exacerbate this issue. In our review, systematic approaches to address adverse events were notably absent. The fact that soft skills and attitudes are often overlooked in surgical competencies poses a challenge for both research on surgical learning and the development of informed surgical education.

Recently, high incidences of burnout among surgery residents have been reported [84]. This concerning trend underscores the need for a holistic approach to surgical education. Addressing stressors in surgical education is not solely an individual concern but a systemic issue, necessitating substantial transformations in healthcare delivery and success measurement [85]. Fortunately, there has been a noticeable increase in publications emphasizing the acquisition of non-technical skills, reflecting a growing awareness of their importance in surgical training [86]. However, it is essential to note that most literature on simulation-based surgical training still predominantly focuses on technical skills [86]. This ongoing emphasis suggests that while strides are being made towards a more comprehensive educational approach, there remains a significant skew towards technical proficiency in current training paradigms.

The studies we reviewed applied various validated assessment tools. In this systematic review, learning was most focused on technical skills and evaluated by OSATS or a derivative. OSATS is a validated evaluation tool used for technical skill assessment [87]. While it is the gold standard in evaluation, it has limitations. The use of OSATS is limited in clinical operating room settings. Hence many studies have attempted to optimize and modify it according to their specific needs [32, 88, 89]. An assessment tool must meet the following requirements: (1) the inter-rater reliability must exceed 0.90, and (2) this reliability should be based on the amount of agreement between the observers [90]. Based on Groenier et al.’s systematic review and meta-analysis, considerable caution is required with the use of assessment tools, especially when high-stake decision-making is required [91]. Advancing proficiency in technical skills with progression toward clinical application poses many issues. Surgeons gaining false self-confidence through inadequate testing may increase the risks of adverse events in clinical applications. Thus, competence testing protocols must be validated, and must be evidence based. In addition to technical proficiency, a surgical intervention requires vast competence and robust, validated assessment tools for surgical soft skills, including learning and interpersonal skills and attitudes.

The results showed that learning by doing, typically simulation, and learning through guided reflection were the most used instructional methods to promote surgical residents’ and medical students’ surgical learning. Both methods effectively promote acquiring knowledge about performing surgical tasks and surgical skills. For instance, simulation training has been shown to enhance fluency in technical performance of specific surgical procedures and patient safety and in increasing a surgeon’s confidence [17, 51, 91]. While building confidence is essential for progression, self-reflection to maintain competence awareness is needed. Hence, self-assessment is fundamental to surgical learning and can be used in many forms [92]. Also, modeling, particularly observing more experienced surgeons performing surgical procedures, self-directed study, and direct instruction were commonly applied to enhance surgical learning. In turn, learning by gaming and mentoring or teaching fellow training surgeons was rarely applied in the studies as forms of instruction in cultivating surgical learning. The result indicates that gaming and peer learning are still both under-studied and under-utilized resources for systematically promoting the learning of future surgeons. The quality and quantity of social interactions with peers, senior surgeons, and patients are fundamental for surgical learning. Learning of all higher-order competencies proceeds from an inter-individual to an intra-individual sphere [93–95]. Moreover, since no surgeon works alone, the surgeon must be trained to work with and within the team. Accordingly, systematic use of peer learning would be essential not only for enhancing specific surgical knowledge and skills, but also for cultivating much-needed surgical soft skills. Nevertheless, emerging qualitative evidence suggests that peer learning is being increasingly implemented in medical education [96]. This trend underscores the growing recognition of the value of collaborative learning environments, where peers can share knowledge, challenge each other, and collectively develop the comprehensive skill set required in modern surgical practice.

Half of the studies we reviewed applied multimodal instruction to enhance surgical learning. This reflects a more modern understanding of learning in which varied instructional methods should be used depending on the object of learning, participants, and context. It also implies that traditional surgical teaching methods of incremental responsibility, with increasing volume-based exposure during residency, will gradually complement more varied research-informed instructional practices. However, it is essential to recall that learning always depends on our actions. This means that if we want to educate reflective practitioners who are good at solving complex problems [36], able to work in teams and engaged in continuous professional development, the instructional designs must systematically engage the future surgeons in such activities [97].

However, based on our review, many questions remain unanswered. The most fundamental of these is related to the transfer of surgical learning from a learning setting to other settings and across the competence ingredients. Firstly, further studies are needed on the extent and how surgical competencies, particularly beyond the technical skills attained in simulation (for instance), transfer into clinical work. This is also connected with the optimal length of the interval between preparation and execution, which was not analyzed thoroughly in most articles, nor was the time for initiation of skill waning explicitly stated. Feins et al. observed a transient decline from the end of one session to the beginning of the next, which was subsequently recovered and improved [37]. Green et al. showed that technical skills attained during preparatory courses are maintained into residency without additional interventions, with similar results from Maertens et al. and Lee-Riddle et al., who recorded proficiency levels to be maintained for at least three months [41, 51, 60]. Secondly, based on our review, studies addressing the learning and training of surgical competencies were highly task specific. Accordingly, further studies on the interrelation between competence ingredients, including surgical knowledge, technical and soft skills, and attitudes, are needed to promote the development of comprehensive surgical competencies among future surgeons. Thirdly, while simulation has proven essential for technical training, many operative interventions contain elements that cannot be simulated with current systems. The preparation for such interventions demands a multimodal approach, including preparatory discussions and visualization, until further methods become available.

Surgical residency is demanding in many aspects, not the least timewise. Among surgeons, mini-fellowships are uncommon as a learning method as opposed to traditional learning-by-doing approaches. While more effective methods are acknowledged, they are not applied due to time concerns [98]. As shown by Bohl et al., dedicated synthetic model training may alleviate time demands, allowing residents to recover better and thus improving preparedness for subsequent tasks [45]. Cognitive task analysis-based training is a valuable adjunct to the modern surgical curriculum, especially considering the global reduction in operating times and volumes during training [99, 100]. CTA-based training improves procedural knowledge and technical performance [99]. However, it was applied in only a few of the studies analyzed here. Interestingly, CTA seems more effective in the later stages of surgical education, with less impact on medical students [101]. In addition, CTA-based training is suitable for electronic delivery, utilization through web-based tools, and gaming applications, all of which are accessible and provide opportunities for frequent revisits without personnel or resource investments [102, 103]. Learning through gaming was also rarely applied in teaching situations in the studies analyzed here. While serious gaming in medical education is beneficial, validating each application for a specific purpose is mandatory [104].

Postgraduate medical education has recently moved towards competency-based education in many countries. Entrusted professional activities (EPA) are utilized as milestones in many competency frameworks [105]. Although EPAs have been applied to and gained rapid acceptance in postgraduate medical education, their potential within undergraduate education remains unverified [106]. In addition, while EPAs are becoming more prominent in surgical education, their widespread adoption and dissemination remain challenging [107]. We advocate for using all tools that collectively embrace a holistic approach to all competency components within surgical learning.

Our study is not without limitations. While we attempted to acquire a comprehensive picture of the pedagogical surgical landscape, we may have yet to detect some reports. Although geographical coverage was acceptable, all the studies we identified were from Western countries. Thus, the actual coverage of multimodal surgical learning warrants further studies. One potential limitation of our study is the decision to restrict our literature search to studies published from 2017 onwards. While this approach allowed us to focus on the most recent and relevant developments in surgical training and competence, it may have excluded earlier studies that could provide additional historical context or foundational insights into the evolution of surgical education practices. Finally, although we limited our study population to students and residents, learning continues through a surgeon’s career and evolves depending on the learner’s situation. Competence-based learning applies equally to all stages of surgical learning and should be incorporated, irrespective of career stage.

## Conclusion

Advancing proficiency through adequate competency assessment is crucial for effective surgical learning. As we observe, contemporary surgical education is high quality and continuously evolves. Most studies focused on objective assessments, yet the measurement and assurance of the transition from technical to clinical proficiency remain areas for further exploration. Defining competency and creating validated assessments are fundamental to lifelong surgical learning.

While acquiring operational skills, decision-making knowledge, and confidence in performing technical tasks are teachable, the ultimate success in learning also hinges on the learner’s attitude and willingness to learn. Therefore, it is vital to incorporate non-technical skills alongside technical aptitude testing and academic achievements in designing modern surgical curricula.

To optimize learning outcomes, learners must adopt an approach encompassing the full spectrum of surgical education. This means integrating technical and non-technical skills to create a learning environment that nurtures a broad range of competencies essential for comprehensive surgical expertise.

## Data Availability

The dataset supporting the conclusions of the current study is available from the corresponding author on reasonable request.
